# BEYOND PHYSICAL ABILITY: PROMOTING INCLUSION IN DEFINITIONS OF SUCCESSFUL AGING

**DOI:** 10.1093/geroni/igae098.3536

**Published:** 2024-12-31

**Authors:** Payton Rule, Emily Willroth

**Affiliations:** Washington University in St. Louis, St. Louis, Missouri, United States; Washington University in St. Louis, St. Louis, Missouri, United States

## Abstract

Physical function and the absence of disability have long been conceptualized as key components of successful aging in most frameworks. However, prior work in disability studies and psychology have suggested that physical functioning is not necessary for successful aging and that this conceptualization may be ableist in nature. Specifically, this definition implies that people with congenital disabilities are not able to age successfully and reinforces society’s view that able-bodied lives are better, happier, or more worthy than disabled people’s lives. This stands contrary to prior research demonstrating high engagement and wellbeing among individuals with disabilities. Moreover, this definition fails to acknowledge the role of inclusive environments (e.g., no stairs, wide doors/walkways) in promoting participation and successful aging. All these factors may lead to increased disability shame, stigma, and discrimination, which can decrease wellbeing, health, and longevity for people with disabilities. In this conceptual presentation, we draw from research in psychology, gerontology, and disability studies to suggest a broader and more inclusive definition of successful aging as continued engagement in activities one needs and wants to participate in. We describe how this definition promotes inclusion by acknowledging that participation can take many forms (e.g., through adaptive devices) and recognizing the power of the environment for promoting successful aging. In this way, we argue that this definition of successful aging takes the onus of functioning off the individual and places it on society to create inclusive environments, allowing all individuals the potential to age successfully, regardless of physical ability.

